# Dr. Abraham Stout

**Published:** 1858-01

**Authors:** 


### NECROLOGICAL NOTICES.

DR. ABRAHAM STOUT was born in Williams Township, Northampton County, Pennsylvania, on the 14th of February, 1793. His home was situated in a beautiful valley, among the Kittatinny Hills, which would have delighted the eye and taste of a Shenstone. He remained at home until he entered the drug-store of the late Dr. Fickardt, of Easton, from whence he went to Philadelphia, where he studied under Dorsey and Physick, whose friendship and confidence he retained as long as they lived. During that time he matriculated in the University of Pennsylvania, and after a careful attention to the studies prescribed, graduated in April, 1819. Two days after, he married Anna Maria, daughter of the late Asher Miner, Esq., of Doylestown, Pa.

He now settled at Springtown, a few miles from his native place; but after having been there a short time only, he was invited by the Moravian hierarchy to remove to Bethlehem, where he resided, with brief intermissions, until a very few months before his death, which took place on the 14th of March, 1857.

On the 5th of January, 1855, he lost his wife, a lady of unusual intelligence and excellence of character, whose biography, could it be written, would, perhaps, be as interesting as his own. This loss affected him deeply, and for a time seemed to disarm him for exertion.

Dr. Stout commenced the thirty-six years of his practice with indefatigable industry. Modest, persevering, and eminently industrious, his every energy was concentrated upon success in the profession he had chosen. His ambition was never of a monetary nature; indeed his attention was never thus engaged save when his desire to educate his children, or his profuse hospitality obliged him to attend to his fiscal affairs.

His success as a medical practitioner was great, and few country surgeons ever performed so many critical and trying operations with the same happy results. His manner to the sick was peculiarly kind and winning; his presence always gave pleasure to the patient, and by his cheering and encouraging words he often brought a cure which medicine alone might never have succeeded in affecting.

His specialties were the epidemic and endemic diseases of Northampton County, Asiatic Cholera, and the diseases of children. He prepared a valuable paper on these diseases, which, together with its geological charts and thermometrical tables, is deposited among the archives of the Northampton County Medical Society.

Surgery was his favorite branch. He was ambidexter, and in amputating a limb, or in operating for cataract, used his hands alternately without any difficulty.

He was a skillful lithotomist, as many can testify. Once he removed from the bladder of a female a calculus as large as an egg. From its apex protruded two-thirds of a darning-needle, the eye-end of which was so firmly imbedded in the specimen that it was found impossible to remove it without destroying the stone.

Hernia, in strangulated cases, presented no impediment to him; and he often subjected cancerous ulcers of the most virulent and painful character to the scalpel.

He was bold, thorough, and decided in performing operations, and, with but few exceptions, always met with remarkable success.

Dr. Stout was a handsome man, of fine physical development, and delicate features. He was extremely domestic, passing much of his leisure with his family. Very hospitable and liberal, he kept an open house, and had a hearty welcome for all; and, though but little of an epicure himself, his table was always well supplied with seasonable delicacies and viands of the choicest and most delicious flavor.

He cultivated the Catawba and Isabella grapes to a great extent, and in one year made twenty barrels of wine from them. This business is still continued at Bethlehem, and Reading, and also at Cincinnati. He also manufactured champagne wine extracted from the white currant, which in quality equalled the best approved foreign brands.

He was very fond of natural science, and in early life spent much of his time in collecting specimens in entomology, mineralogy, and botany.

The death of Dr. Stout caused profound grief in the social and professional circles of which he had so long been an ornament. His funeral obsequies were attended by a large number of mourning and sympathizing friends, who felt how difficult it would be to replace the loss they had sustained. Endowed with superior talents and a noble heart, his life was replete with laudable generous actions, which will ever live in the memories of those who knew him.

His remains were interred in the Moravian cemetery, in a spot which he had himself chosen for the home of his last dreamless sleep. M. R. S.

